# Smooth Emergence from General Anesthesia after Deep Extubation in a Pediatric Patient Diagnosed with Catecholaminergic Polymorphic Ventricular Tachycardia: A Case Report

**DOI:** 10.3390/medicina59122067

**Published:** 2023-11-23

**Authors:** Seung Bae Cho, Beomseok Choi, Seunghee Ki, Seokwoo Hwang, Juseok Oh, Insik Jung, Jeonghan Lee

**Affiliations:** Department of Anesthesiology and Pain Medicine, Busan Paik Hospital, Inje University College of Medicine, Busan 47392, Republic of Korea; 089517@paik.ac.kr (S.B.C.); 089535@paik.ac.kr (B.C.); 108873@paik.ac.kr (S.K.); 094157@paik.ac.kr (S.H.); 093724@paik.ac.kr (J.O.); 094462@paik.ac.kr (I.J.)

**Keywords:** anesthetic management, arrhythmia, catecholaminergic polymorphic ventricular tachycardia, endotracheal extubation, intraoperative monitoring, postoperative nausea and vomiting

## Abstract

*Background:* Catecholaminergic polymorphic ventricular tachycardia (CPVT) is a rare genetic disorder where catecholamine causes bidirectional ventricular tachycardia, potentially leading to cardiac arrest. In patients undergoing surgery, sympathetic responses can be triggered in situations associated with surgical stimulations as well as high anxiety before the surgery, anesthetic maneuvers such as endotracheal intubation and extubation, and postoperative pain. Therefore, planning for surgery demands meticulous attention to anesthesia during the perioperative period in order to prevent potentially life-threatening arrhythmias. *Case:* We discuss a case of an 11-year-old male pediatric patient with known CPVT who required elective strabismus surgery for exotropia involving both eyes. After thorough planning of general anesthesia to minimize catecholamine response, sufficient anesthesia and analgesia were achieved to blunt the stressful response during intubation and maintained throughout the surgical procedure. Complete emergence was achieved after deep extubation, and the patient did not complain of pain or postoperative nausea and vomiting. *Conclusions:* Anesthesiologists should not only be able to plan and manage the catecholamine response during surgery but also anticipate and be prepared for situations that may lead to arrhythmias before and after the procedure. In certain cases, deep extubation can be beneficial as it reduces hemodynamic changes during the extubation process.

## 1. Introduction

Catecholaminergic polymorphic ventricular tachycardia (CPVT) is a rare genetic disorder that affects the cardiac conduction of a structurally normal heart [1]. Clinical manifestation of CPVT in most cases is syncope or sudden cardiac death that is triggered by physical activity or emotional stress that increases sympathetic activity. The increased catecholamine leads to excessive calcium release in the heart, thereby causing arrhythmia that manifests as polymorphic or bidirectional ventricular tachycardia (VT), potentially leading to cardiac arrest [2,3]. Therefore, in the management of anesthesia in patients with CPVT, preventing sympathetic activation that triggers arrhythmia is of utmost importance, and methods to reduce stress during anesthetic and surgical manipulations should be carefully planned in order to minimize catecholamine release. This case report describes the anesthetic management of an 11-year-old pediatric patient with known CPVT scheduled for strabismus surgery. Sufficient anesthesia and analgesia were maintained throughout the procedure, and complete emergence was achieved after deep extubation.

## 2. The Case Description

An 11-year-old male (height 148 cm, weight 45 kg) pediatric patient was referred to our hospital for strabismus surgery for exotropia involving both eyes accompanied by aggravating diplopia and headache. His past medical history includes syncope during strenuous exercise, and he was diagnosed with CPVT 3 years ago at another hospital. Since then, he has not participated in physical exercises and avoided stressful activities. He was medicated with nadolol 20 mg twice daily and underwent regular checkups with a pediatric cardiologist. An implantable cardioverter defibrillator (ICD) was not inserted because there were no recurring episodes of loss of consciousness or cardiac arrest. He did not have any other underlying disease, and the only other recent medication was acetaminophen for the aggravating headaches. 

**Figure 1:**
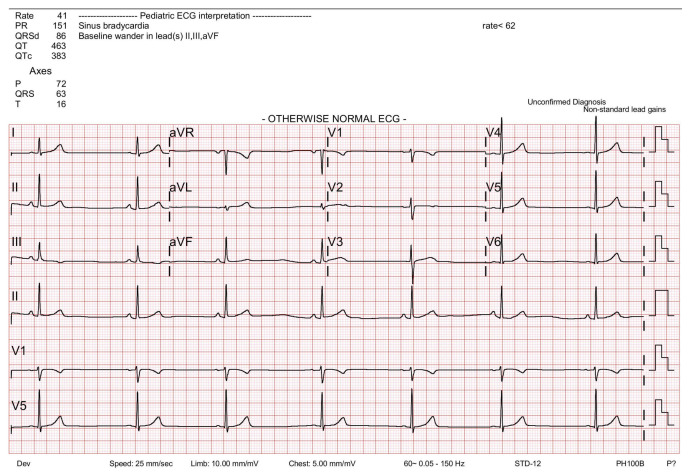
Electrocardiogram (ECG) taken as a routine pre-operative evaluation shows sinus bradycardia with heart rate of 41 beats/min.

The patient underwent routine pre-anesthetic evaluations, including blood laboratory tests and chest radiography. Other than sinus bradycardia of heart rate (HR) 41 beats/min recorded in both the electrocardiogram (Figure 1), all other lab values and chest radiography were within normal range, and physical examination did not reveal any abnormalities related to the cardiovascular system. After a comprehensive review and careful planning of anesthesia, the pediatric patient was approved for general anesthesia and admitted to the pediatric ward one day before the scheduled surgery. 

On the day of surgery, the patient took his usual morning dose of nadolol 20 mg. Upon arrival in the operating room, non-invasive blood pressure (BP), electrocardiogram (ECG), oxygen saturation (SpO_2_), bispectral index (BIS), and electromyogram were monitored. Automated external defibrillator (AED) pads were applied above the right nipple below the clavicle and on the left axilla at the fifth intercostal space in the event of ventricular tachycardia and attached to M4735A HeartStart XL Defibrillator (Philips Medical Systems, Andover, MA, USA) under manual mode. The initial vital signs were all within normal range: BP 109/64 mmHg, HR 46 beats/min, SpO_2_ 99%, and body temperature (BT) 36.5 °C. Subsequently, atropine 0.25 mg and dexamethasone 2.5 mg were administered intravenously. While delivering 100% oxygen at 10 L/min for 5 min via facemask, remifentanil (0.5 μg/kg/min) was continuously infused, followed by propofol 100 mg for the induction of general anesthesia. After confirming that the BIS values had dropped to 34, rocuronium 30 mg was administered. Facemask ventilation was easy without excessive pressure. 

An arterial line was placed in the left radial artery, and after confirming deep neuromuscular block via train-of-four (TOF), intubation was performed with a 6.0 mm internal diameter (ID) reinforced endotracheal tube (ETT) using a video laryngoscope. The BP measured immediately after intubation was 91/46 mmHg, and the HR was 79 beats/min, which was the fastest HR observed throughout the entire surgical and anesthetic procedure. Anesthesia was maintained with 2.2% sevoflurane and remifentanil (0.05 μg/kg/min). The lungs were mechanically ventilated using the volume-controlled mode of the Dräger Primus anesthesia workstation (Dräger Medical, Lübeck, Germany) using a closed circuit with the following settings: tidal volume of 8 mL/kg, respiratory rate of 18-20 per min, FiO_2_ 0.5, and fresh gas flow of 3 L/min. Subten block was performed by the ophthalmologist after anesthetic induction using 0.2% ropivacaine 2 mL to both eyes. There were no significant changes in the vital signs throughout the surgical procedure. Approximately 15 min before the end of the surgery, the continuous infusion of remifentanil was stopped, and simultaneously, dexmedetomidine (0.5 μg/kg) was administered over 10 min. Ondansetron 4 mg, ketorolac 15 mg, and acetaminophen 500 mg were administered for pain control and prevention of postoperative nausea and vomiting (PONV).

**Figure 2:**
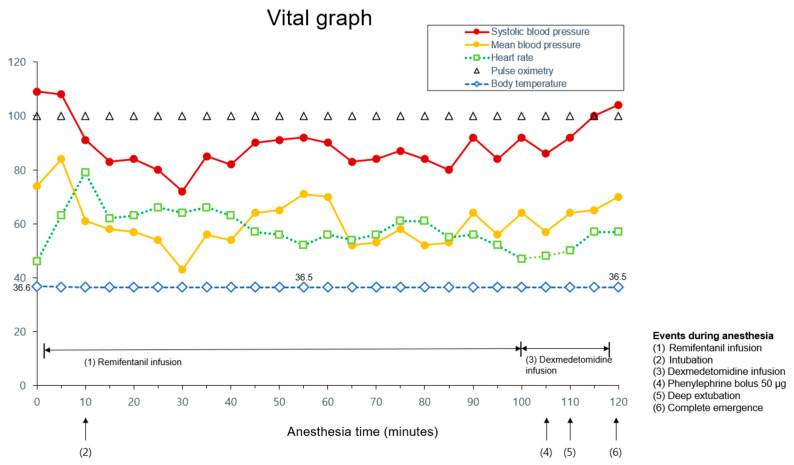
Graph of vital signs of the pediatric patient recorded in the operating room. Events during anesthesia are marked with arrows. Maximum heart rate of 79 beats/min was recorded immediately after endotracheal intubation. Phenylephrine bolus injection of 50 μg was given when the mean arterial pressure was 57 mmHg during dexmedetomidine infusion period when there were no surgical stimuli.

Once the surgery was nearly complete, steps were taken to perform a deep extubation. Muscle relaxation was reversed with sugammadex (2 mg/kg) and confirmed by a TOF ratio above 0.9. Sevoflurane remained unchanged at 2.2% in order to maintain 1 minimum alveolar concentration (MAC). Oral secretions were aspirated, and a lack of response to oropharyngeal stimulation was confirmed. We waited for spontaneous breathing to return until tidal volumes greater than 5 mL/kg were achieved without any mechanical assistance. After preparation had been made for a re-intubation situation in the event of airway obstruction, the ETT was carefully extubated. The BIS value at the time of extubation was 43. After extubation, 100% oxygen was supplied via a facemask, and spontaneous breathing returned within 15 s. Anesthetic agents were cleared with a fresh gas flow rate of 12 L/min. The patient’s consciousness was fully restored in less than 10 min, during which there were no signs of respiratory distress or emergency delirium. The vital signs when spontaneous breathing returned were BP 104/53 mmHg, SpO_2_ 98%, HR 57 beats/min, and BT 36.5 °C. The maximum HR from the start to the end of anesthesia was 79 beats/min, and no ventricular arrhythmias were observed during the 2 h procedure (Figure 2).

The patient was transferred to the recovery room, where continuous monitoring of ECG, BP, SpO_2_, and HR were conducted. He remained alert and did not complain about PONV. Oxygen saturation remained above 97% without supplemental oxygen, and vital signs were confirmed to be within 20% of the pre-anesthetic levels. The patient was transferred to the pediatric ward and discharged two days after the surgery without any cardiovascular events or complications related to the surgery. 

## 3. Discussion

CPVT is a rare genetic disorder with an estimated prevalence of 1 in 10,000 individuals, characterized by life-threatening arrhythmias in individuals with structurally normal hearts [1]. The most common type of CPVT is type 1, caused by mutations in the RyR2 gene [1]. Patients with CPVT experience episodic syncope occurring when sympathetic nerve stimulation increases in situations such as physical exercise or emotional stress. The underlying cause of these episodes is arrhythmia that manifests as polymorphic or bidirectional ventricular tachycardia (VT), triggered by the discharge of catecholamines via sympathetic nerve stimulation [2,3]. The discharge of catecholamine triggers the mobilization of calcium ions (Ca^2+^), leading to an elevation in the intracellular Ca^2+^ concentration within cardiomyocytes, thereby inducing arrhythmias [3]. Although spontaneous resolution of arrhythmia is possible, the absence thereof may exacerbate ventricular tachycardia, degenerating into ventricular fibrillation and ultimately precipitating sudden cardiac arrest. Approximately 30% of individuals with CPVT encounter at least one cardiac arrest, and up to 80% experience one or more syncopal episodes [2]. The treatment of CPVT aims to prevent life-threatening arrhythmias. Management typically involves the use of beta-blockers, and flecainide may be added to the treatment regimen [1,2]. In cases where medication therapy is insufficient to control the occurrence of arrhythmias, additional options such as implantable cardioverter defibrillator (ICD) or left cardiac sympathetic denervation (LCSD) may be considered [1,2].

The anesthetic management of CPVT patients should aim to prevent catecholamine release to avoid arrhythmias and be prepared in advance for prompt intervention in case of arrhythmia occurrence. Therefore, one should consider situations where catecholamine release can occur not only during surgery but also before and after surgery. We planned the anesthesia procedure of our patient with the following objectives: (1) to manage the patient’s stress due to preoperative anxiety or surgery-related concerns, (2) to maintain a sufficient depth of anesthesia in order to minimize hemodynamic response to surgical stimuli, as well as to be prepared for intraoperative arrhythmia occurrence, and (3) to effectively control postoperative pain to reduce pain-related stress.

Because it is important to continue taking beta-blockers before and after surgery to prevent arrhythmias in CPVT patients, we instructed the patient to take a beta-blocker on the morning of the surgery [4]. Considering the potential sympathetic trigger during procedures such as intravenous catheter insertion, as well as the patient’s preoperative anxiety related to surgery, we considered medicating anxiolytic drugs to the patient. Benzodiazepines, such as midazolam or temazepam, can safely reduce anxiety and the stress associated with surgery and procedures in CPVT patients [5,6]. However, since the patient did not experience significant stress during previous intravenous catheterizations and did not exhibit anxiety on the day of surgery, an anxiolytic drug was not administered. Instead, we chose to continuously monitor the patient’s ECG, SpO_2_, and BP after admission to the pediatric ward in order to promptly detect any arrhythmias. Therefore, we performed intravenous catheter insertion while the patient was under monitoring and continued monitoring until the patient was moved to the operating room. During the transition to the operating room, we allowed the parents to accompany the patient for emotional support. 

In general anesthesia, surgical and anesthetic manipulations can lead to various hemodynamic changes in patients. The anesthesiologists should consider the hemodynamic responses resulting from these surgical stimuli and proceed with the anesthesia accordingly. The choice of anesthetic agents in CPVT patients should be carefully considered to avoid causing arrhythmias. Ketamine is commonly used for general anesthesia in pediatric patients but is not suitable for CPVT patients due to its stimulation of sympathetic nervous system activity [6,7]. Sevoflurane, propofol, and remifentanil can be safely used for the induction and maintenance of anesthesia in CPVT patients [4,7]. Rocuronium, like other neuromuscular blocking agents, with the exception of pancuronium, can be safely used in CPTV patients [4]. Hypotension can occur during general anesthesia as a result of beta-blocker therapy, and positive-pressure ventilation can exacerbate this by reducing venous return [4,5]. The treatments for hypotension in CPVT patients include the Trendelenburg position, appropriate fluid administration, and the use of pure α-adrenergic agonists such as phenylephrine [4,5,7]. Hypotension caused by severe bradycardia should be treated with atropine or pacing [5,7]. However, β-adrenergic agonists should be avoided as they may trigger arrhythmias [4,5,7].

In the event of VT that triggers the need for cardiopulmonary resuscitation (CPR), treatment should be specific for the individual with CPVT. The persistence of polymorphic ventricular ectopy or the presence of bidirectional VT is suggestive of such dysrhythmic storms associated with CPVT, and in these patients, epinephrine is contraindicated even during the arrest phase because it can contribute to the maintenance of VT storms [8]. Furthermore, the depth of anesthesia should not be compromised in order to improve hemodynamic values. After resuscitation, immediate focus should be made to suppress the dysrhythmic storms using approaches to counteract the sympathetic activations using beta-blockers and opioid administrations [7,8]. In our case, we had prepared the following to be immediately utilized in the event of CPR: (1) defibrillation via attached AED pads (4–8 J/kg); (2) intravenous beta-blocker therapy (esmolol 500 μg/kg bolus); (3) remifentanil bolus administration via attached infusion pump; (4) magnesium administration (50 mg/kg); and (5) amiodarone administration if needed (5 mg/kg).

Endotracheal intubation is a crucial procedure that requires careful attention in the anesthetic management of CPVT patients as it elevates the patient’s plasma catecholamine levels and induces subsequent hemodynamic responses [9]. To avoid hemodynamic changes, we initially considered the use of a laryngeal mask airway (LMA) instead of ETT. LMA insertion in pediatric patients is associated with less hemodynamic response compared to endotracheal intubation [10]. Furthermore, the removal of LMA is associated with fewer hemodynamic changes compared to extubation of ETT after surgery [11,12]. Considering these factors, it appears that using LMA may offer advantages over ETT in achieving the objective of minimizing hemodynamic responses. However, while planning for anesthesia, we discovered that the patient’s tooth number 53 was mobile when we examined our patient’s teeth. While dental damage due to LMA is not common, there is a risk of inadvertent tooth loss if the patient bites down on the LMA with mobile teeth [13]. Considering the potential stress, the risk of bleeding, and the possibility of aspiration of lost tooth or blood due to dental damage, we decided to forgo the use of an LMA and perform intubation with an ETT. We applied AED pads and secured an arterial line before performing endotracheal intubation to prepare for potential arrhythmias that might occur due to catecholamine release induced via endotracheal intubation. We performed intubation smoothly without teeth damage and secured it in place with a bite block to prevent the patient from biting down on the ETT with the mobile tooth. Following the intubation, the patient’s HR reached its highest at 79 beats/min, the fastest heart rate recorded during the surgery. Fortunately, no arrhythmias occurred; however, we were concerned about the potential catecholamine release during the process of extubation. Therefore, we planned for a deep extubation to achieve a smooth extubation. 

Smooth extubation refers to an extubation maneuver that maintains acceptable ventilation without causing patient discomfort or significant changes in vital signs [14]. The methods for achieving smooth extubation include deep extubation, exchanging ETT for an LMA, and ‘No-Touch’ extubation, where no stimulation is allowed until patients spontaneously wake up [14]. Deep extubation may reduce hemodynamic complications like tachycardia and hemodynamic swings [14]. Also, a recent systemic review and meta-analysis showed that deep extubation, compared to awake extubation, does not increase the risk of laryngospasm and breath holding while reducing the risk of overall complications, including cough and desaturation [15]. Pharmacologic intervention of intravenous dexmedetomidine can facilitate deep extubation. Intravenous dexmedetomidine can support smooth extubation without the added risk of respiratory depression and decrease agitation and pain scores and the amount of severe coughing [7,14]. In previous case reports regarding anesthesia in CPVT patients, dexmedetomidine was safely used for sedation [3,7]. Despite these advantages, deep extubation with pharmacologic intervention should not be routinely applied to extubation in CPVT patients. The administration of dexmedetomidine may exacerbate preexisting bradycardia and hypotension in patients with beta-blocker therapy [16]. Furthermore, deep extubation can increase the risk of airway obstruction, which poses a threat to patient safety [15]. Therefore, before planning deep extubation, anesthesiologists should carefully weigh its advantages against awake extubation, consider clinical factors that might negatively impact patient ventilation post-extubation, and establish definitive plans in cases when maintaining adequate ventilation post-extubation becomes challenging [17]. 

In our case, given that the patient did not have a difficult airway and considering the importance of minimizing hemodynamic changes during the extubation process, we chose deep extubation. We also contemplated ‘No-Touch’ extubation but ultimately ruled it out due to concerns about patient agitation stemming from stimulation caused by the ETT before the patient fully emerged from anesthesia. Instead, after performing deep extubation, we confirmed the patient’s adequate spontaneous respiration and then limited stimulation to maintain the advantages of ‘No-Touch’ extubation. Deep extubation was performed by an experienced specialist with the medications and equipment readily available in the event of airway obstruction or unstable vital signs. After the administration of dexmedetomidine, the patient experienced a decrease in both heart rate and blood pressure. The patient’s heart rate was low but did not further decrease compared to the initial reading, so the condition was closely observed. Phenylephrine bolus (50 µg) was injected during the dexmedetomidine infusion period when there were no surgical stimuli when the blood pressure was 86/43 mmHg, and the heart rate was 48 beats/min. 5 min after injection, the blood pressure and heart rate changed to 92/50 mmHg and 50 beats/min, respectively.

Traction on the extraocular muscle or other ocular interventions can stimulate the vagal center, leading to the occurrence of the oculocardiac reflex (OCR) [18]. OCR is defined as bradycardia or arrhythmia during ocular surgery, with a reported prevalence of up to 90% [18]. The bradycardia resulting from OCR poses a risk of severe bradycardia in patients receiving beta-blocker therapy. Premedication with anticholinergics may be helpful in preventing OCR, but complete prevention is challenging [18]. Subtenon block is a commonly used technique in ophthalmic surgery. Subtenon block can reduce postoperative pain, decrease the incidence of OCR and vomiting, and help reduce the need for additional analgesics [19]. Recognizing that these benefits can be applied to our patient, the subtenon block was performed by an experienced and skilled ophthalmologist, who has more expertise in this procedure than our anesthesia team. Our patient did not experience a significant decrease in heart rate during surgery, did not complain of PONV, and did not report postoperative pain, suggesting that subtenon block may have contributed to these outcomes. Postoperative pain in CPVT patients poses a risk of arrhythmia, and the use of opioids for additional analgesia is a factor that can induce PONV [20]. Therefore, when planning general anesthesia for CPVT patients, consideration should be given to incorporating regional anesthesia to mitigate these risks. There are no specific contraindications to the use of local anesthetics in CPVT patients. In other studies, ropivacaine was safely used in serratus plane blocks for CPVT patients [16], and bupivacaine was safely used in combined spinal-epidural anesthesia [21]. However, it is contraindicated to mix local anesthetics with epinephrine [4].

A high fresh gas flow of 5 L/min or more is commonly used for emergence from inhalation anesthesia [22]. We used a very high fresh gas flow of 12 L/min for emergence from inhalation anesthesia. Higher fresh gas flow helps in the rapid removal of volatile anesthetics within the breathing circuit and prevents the rebreathing of the patient’s exhaled air [23]. We believed that this would aid in the quick elimination of volatile anesthetics from the patient and shorten the time to emergence. Since volatile anesthetics are a major cause of PONV in patients, rapid removal of volatile anesthetics can reduce postoperative stress [24]. However, recent studies have shown that increasing the fresh gas flow to 4 L/min or more does not expedite patient emergence [23,25]. When the fresh gas flow was 4 L/min or more, the end-tidal concentration of volatile anesthetics at eye opening and extubation did not significantly differ from other groups with a higher fresh gas flow [23,25]. There was also no difference in PONV occurrence between the two groups [23]. This is likely the result of modern anesthesia ventilator capability to almost completely prevent rebreathing with a fresh gas flow of 4 L/min or more [26]. Considering this, our use of a very high fresh gas flow of 12 L/min for emergence from inhalation anesthesia appears to be excessive. Additional research may be necessary, but increasing the fresh gas flow appears uneconomical and not beneficial for the patient. A low fresh gas flow of 2 L/min or less can reduce the unnecessary use of gas, providing economic advantages [23]. However, emergence time may increase compared to a fresh gas flow of 4 L/min [23].

## 4. Conclusions

Anesthesia in CPVT patients demands meticulous attention to prevent potentially life-threatening arrhythmias. Anesthesiologists should not only manage the catecholamine response during surgery but also anticipate and be prepared for situations that may lead to arrhythmias before and after the procedure. Deep extubation can be beneficial for CPVT patients as it reduces hemodynamic changes during the extubation process. However, before performing deep extubation, it is essential to assess whether it offers advantages over awake extubation for the patient and to anticipate and prepare for the possibility of airway obstruction.

## Data Availability

The data presented in this study are available from the corresponding author upon reasonable request. The data are not publicly available because of privacy or ethical restrictions.
